# Fracture morphology and ossification process of the keel bone in modern laying hens based on radiographic imaging

**DOI:** 10.1371/journal.pone.0312878

**Published:** 2024-10-29

**Authors:** Páll Gretarsson, Åste Søvik, Ida Thøfner, Randi Oppermann Moe, Ingrid Toftaker, Käthe Kittelsen

**Affiliations:** 1 Faculty of Veterinary Medicine, NMBU–Norwegian University of Life Sciences, Ås, Norway; 2 Under Pelsen AS, Ås, Norway; 3 Department of Veterinary & Animal Sciences, University of Copenhagen, Copenhagen, Denmark; 4 Animalia–The Norwegian Meat and Poultry Research Centre, Oslo, Norway; Nasarawa State University, NIGERIA

## Abstract

Keel bone fractures (KBF) are one of the most important welfare problems in commercial laying hens. Despite extensive research on the matter, its etiology remains unclear. Studying fracture characteristics in radiographic images can aid in the understanding of the disorder. The aim of the current study was to provide detailed description of fracture characteristics and explore ossification in the keel bone. In this descriptive study, repeated cross-sectional sampling was performed in one commercial laying hen flock. The flock was visited at 11 time points from 17–57 weeks of age (WOA), radiographing 30 laying hens at each visit resulting in altogether 330 unique radiographs. Fracture characteristics and the keel bone’s level of ossification were assessed in each radiograph. In total, 344 fractures were recorded, of which 71.5% were complete and 28.5% were incomplete. Of the complete fractures, 82.9% were recorded as transverse, and 15.9% as oblique. One comminuted and two butterfly fractures were recorded. The caudal third of the keel was the most common area for fractures. Fracture characteristics differed between the different regions of the keel bone; all incomplete fractures in the cranial third appeared on the ventral surface of the keel, whilst the majority of incomplete fractures on the caudal third appeared on the dorsal surface. This indicates that the underlying etiology might differ between the cranial and caudal part. Folding fractures were observed in 18.6% of all the fractures, and occurred in both cranial-, and caudal third of the keel, indicating possible underlying disorders of calcium metabolism. All hens at 32 WOA and older had a fully ossified keel, based on radiographic evaluation. Displacement and soft tissue swelling are common characteristics in fractures of traumatic origin. We found a high frequency of simple fractures, without these characteristics, indicating that non-traumatic causes may be of higher importance than conventional beliefs.

## Introduction

Keel bone fractures (KBF) have recently been described as one of the most important welfare problems in commercial laying hens [1–5], with its high incidence and associated pain [4, 6, 7]. Additionally, it can lead to increased feed intake and reduced egg production, thereby affecting the farmers’ economy [8]. Studies that have examined laying hens at end of lay, by postmortem examination have reported a prevalence above 80% [1–3].

The etiology of KBF remains unclear, despite numerous studies on the matter as reviewed by Toscano et al. [9], also implying the multifactorial nature of the problem. A common hypothesis is that KBF are caused by external trauma from high impact collisions with the housing system [1, 3, 10–13]. However, the contribution of external trauma in KBF etiology is unclear as there are studies reporting high prevalence of KBF in both cage and cage-free systems [3, 14]. A recent study by Thøfner et al. [2] did not find a significant difference between the housing systems. Additionally, another study by Thøfner et al. [15] described KBF histologically in both caged and non-caged laying hens and found indications that external trauma might not be the main causal factor. High egg production is likely a contributing factor to KBF [16]. Toscano et al. [9] discussed additional factors, including early onset of lay and late ossification of the keel bone. Expanding on this, a study from Denmark reported that small hens with an early onset of lay, with a high estimated daily egg weight in the 1^st^ week of lay were at higher risk of developing KBF by the end of the production cycle [2].

Studies exploring fracture morphology can enhance our understanding of the etiology of KBF, as different factors can affect different fracture types, e.g. comminuted or butterfly fractures are commonly caused by a higher force of impact [17, 18]. Radiography is a common diagnostic tool for evaluating fractures and has been used in several studies to assess the keel bone [19–26]. Radiographical scoring systems and protocols for recording the presence of and/or severity of KBF have been developed [19, 20, 24]. Previous longitudinal studies have used radiography to assess fracture healing [23], fracture prevalence at different ages [21] and fracture incidence [22]. Additionally, Baur et al. [22] provided a detailed description of KBF characteristics in their longitudinal study using radiography in quasi commercial conditions; however fracture characteristics like incomplete fractures, degree of displacement and degree of soft tissue swelling were not described [22]. Nevertheless, detailed descriptions of the fracture morphology are scarcely reported. In diagnostic imaging, fractures are typically categorized based on factors such as their location, direction, completeness or incompleteness, number of fracture lines, degree of displacement, and whether they are open or closed. A transparent and uniform reporting of KBF morphology in studies using radiography can enhance the comparability between studies.

In addition to fracture assessment, radiography can also be used to assess the ossification process. Recent studies on keel bone ossification in modern hybrids are lacking; however, a study from 1948 reported that the keel bone is fully ossified when the hen is 40 weeks of age (WOA), based on macroscopic examination [27]. Up until this age, the caudal part of the keel consists of cartilage that gradually ossifies. This means that from onset of lay at around 20 WOA and up until peak performance at about 40 WOA, their keel bone is not fully ossified. The period from 25–50 WOA has been reported as the period where most new KBF appear, implying that most fractures occur before the keel is fully ossified [13, 14, 22, 28]. Indeed, the direction of the keel’s ossification goes from cranial to caudal, i.e. the caudal third of the keel is the last part to be ossified which is also the most common area for KBF [2, 3, 22, 29, 30]. It is known that the hypertrophic zone in endochondral ossification is a weak point often involved in fractures [31, 32]. However, the impact of late ossification on the development of KBF is not explored and it is therefore important to investigate to improve our understanding of the underlying etiology.

A poor understanding of the mechanisms leading to this important welfare problem hampers the implementation of adequate interventions and preventive measures. Providing a detailed description of the radiographic characteristics of KBF could aid in the understanding of the underlying etiology. Though conducted previously in a quasi-commercial setting [22], this has not been investigated in a true commercial setting. Additionally, the keel’s ossification alongside the development of KBF has not been explored. Therefore, the aim of this study was to describe the fracture characteristics of KBF, assess KBF occurrence and describe the ossification of the keel bone in different age groups, in commercial laying hens from onset of lay to post peak performance.

## Material and methods

### Study sample and study design

In this descriptive study with repeated cross-sectional sampling, one commercial laying hen flock was investigated at 11 time points from 17 to 57 WOA, with approximately four weeks between visits. An overview of time points for visits and flock characteristics are shown in Table 1. All visits and data collection were performed by the first author (PG). The farm housed one flock of 7500 Dekalb White laying hens, which is the standard flock size in Norway [33]. Hens were kept in an indoor multi-tiered aviary system (Big Dutchman NATURA step 24–21 [34]). The floor area in the barn was 477.67 m^2^ with two aviary rows. Each row was approximately 40 m long and 2.5 m wide. The stocking density was 9 hens/m^2^ in accordance with Norwegian legislation [35]. The flock was fed standard commercial pelleted laying hen feed from Felleskjøpet AS (Kromat) tailored to the different phases in egg production. The house had artificial lighting, with both white and yellow light. Lux measurements were not conducted during the study period, however the farmer used a light management with minimum 3–6 lux from 17 WOA. Onset of lay was 20 WOA.

**Table 1:** Overview of sample weeks, sampled hens’ weight and flock data for each week of visit.

	Weight of sampled hens (kg)							
Week of age	Mean	SD	Flock mean weight per hen (kg)^a^	Flock lay percent	Mean* flock egg weight (g/hen/day)	Mean* flock feed intake (g/hen/day)	Mean* flock water intake (ml/hen/day)	Weekly flock mortality (%)
17	1.31	0.12	1.28	0.1	N/A	61.9	70.3	0.07
20	1.53	0.10	1.49	30.4	N/A	95.7	150.8	0.03
25	1.60	0.09	1.67	99.7	57.2	116.9	206.9	0.04
28	1.66	0.15	1.69	99.2	59.7	115.8	202.3	N/A
32	1.76	0.11	1.70	98.9	N/A	116.7	209.6	0.07
36	1.77	0.15	1.72	99.4	62.7	116.4	229.8	0.01
41	1.72	0.13	1.74	99.2	62.6	117.3	218.3	0.05
45	1.70	0.14	1.74	99.1	62.2	115.9	206.7	0.03
49	1.68	0.14	1.74	98.6	62.5	114.1	194.6	0.12
53	1.75	0.19	1.77	98.9	62.5	115.7	196.0	0.45
57	1.86	0.14	1.77	97.8	62.8	116.9	187.9	1.68^b^

* Mean calculated per week.

^a^ Information provided by the farmer from repeated weighing on digital hanging scales placed in the barn

^b^ *Escherichia coli* outbreak in flock.

At each visit, 30 hens were radiographed using a portable x-ray machine (Poskom Vet20-BT) and a direct digital radiography flat panel detector (Konica Minolta, Aero DR NS3543 mobil). The images were acquired with 3.2 mAs, 50 kV and a focus-film distance of 100 cm. The 30 hens were selected by convenience sampling by the farmer during the morning routine. To ensure a representative sample, the farmer was instructed to select hens from the floor and at different levels in the aviary, through the whole length of the house. The sampled hens were temporarily housed in two steel crates (15 hens each), prior to radiography. Each crate was 124 x 83 x 76 cm, with a hard plastic base covered with wood shavings. This was implemented to prevent the possibility of examining the same hen twice during the same visit. One hen at a time was then carried upright from the crate to the x-ray machine, approximately 3 m away in the anteroom. The hen was then carefully held in an inverted position to induce immobility, for radiographic imaging. The right side of the hen was facing the digital flat panel detector, with the keel bone parallel to the detector (latero-lateral view). After confirming the quality of the radiograph, the hen was weighed and finally released into the flock again. Each radiograph got an identification number unique to each hen and visit. The hens were not physically marked with an identification number. Time duration from collecting a hen from the temporary crate, imaging, weighing, and finally releasing the hen back to the flock was approximately 2 minutes per bird. The hens were not anesthetized for the radiographic imaging [36]. At 10 visits (17–53 WOA), five of the 30 examined hens were euthanized for histological examination of the keel bone (to be described elsewhere). This resulted in 50 hens in total. Prior to euthanasia the birds were sedated with i.m. injection of ketamine 30 mg/kg and medetomidine 0.3 mg/kg. After assuring full effect of the sedation (absence of corneal reflex), the birds were euthanized with i.p. injection of pentobarbital 100 mg/kg.

Legal and ethical approval was provided by the Norwegian Food Safety Authority (approval number 29556). The farm is privately owned by the farmer, and no protected or endangered species were involved in the study. All efforts were made to minimize suffering. The collected hens were observed by a veterinarian during their time in the temporary crates, and the radiological examination. The hens were observed for signs of stress (excessive wing flapping and vocalization, aggression, restless pacing) in the temporary crate and during radiological examination. Signs of stress would result in immediate release back into the flock and exclusion from the study. Hens showing signs of lethargy or trauma were euthanized immediately with the same protocol as described above.

The whole flock was euthanized at 59 WOA due to a severe *Escherichia coli* outbreak (sequence type ST-141, serotype O2/O50:H6), which explains the increase in weekly mortality from 49 WOA (Table 1). Euthanasia of the flock was carried out by introducing CO_2_ into the barn (minimum 45% CO_2_ concentration) as is a standard procedure in the egg industry in Norway.

### Radiographic evaluation

**Fig 1:**
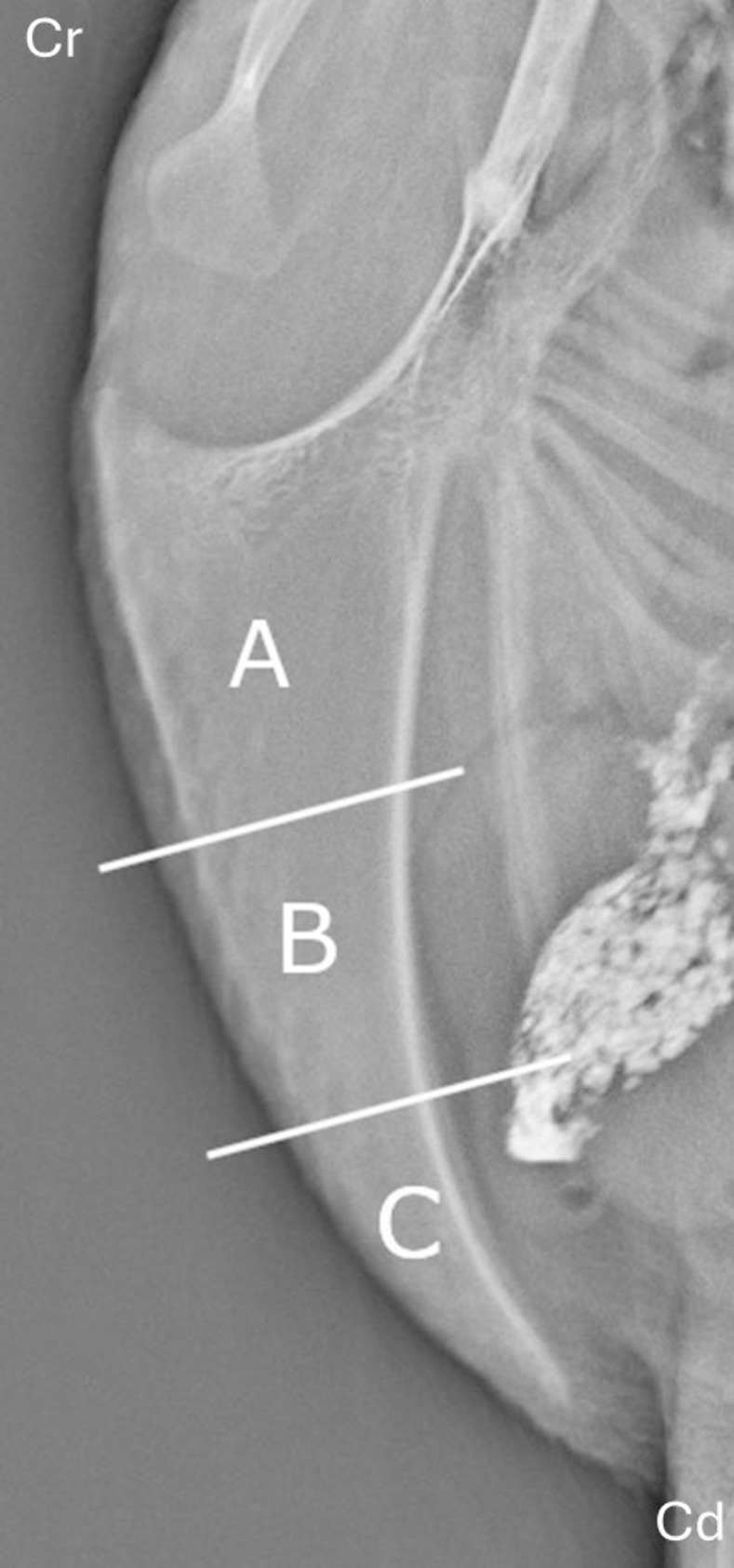
Dividing the keel bone into thirds. Figure showing the keel bone divided into thirds. A = cranial third, B = mid third, C = caudal third. Cr = cranial, Cd = caudal. Radiograph of a 20 WOA hen from the study sample. The keel has no lesions and is not fully ossified. Division of the keel bone adapted from Baur et al. [22] and Thøfner et al. [15].

**Fig 2:**
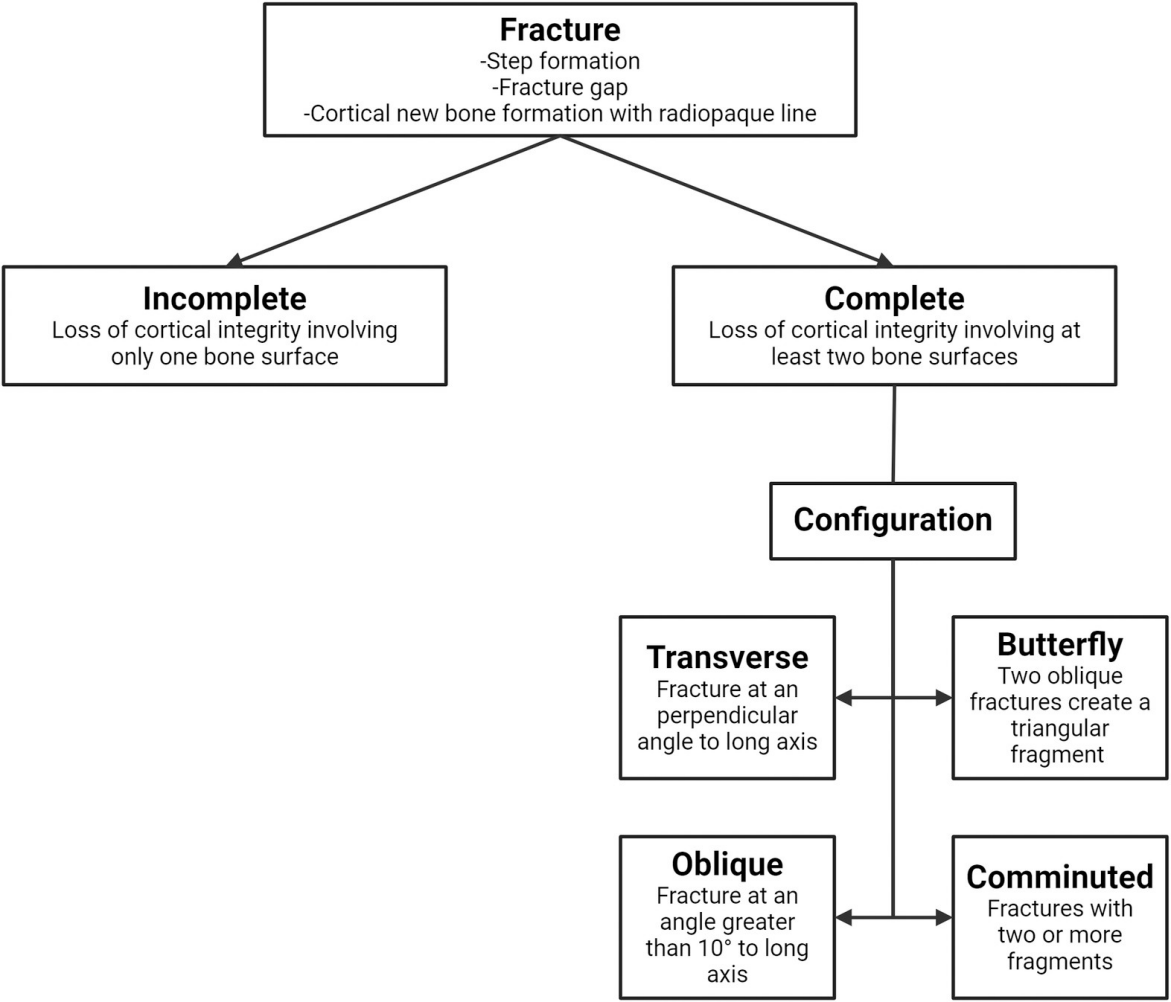
Radiological characterization of fractures. A flowchart of radiological characterization of fractures and criteria for incomplete and complete fractures, as well as their configuration. Only complete fractures were further divided with configuration in the current study. Created with BioRender.com.

The radiographic images were imported into the OsiriX software (DICOM viewer, OsiriX v. 12.5.2, Pixmeo SARL, Switzerland) and evaluated by a single investigator (AS) (Diplomate of the European College of Veterinary Diagnostic Imaging) using MacBook Pro 2020 (Quad-Core Intel Core i5 with Intel Iris Plus Graphics 1536 MB, macOS Monterey v. 12.6.3). Criteria for radiographic evaluation regarding dividing the keel bone into thirds, fracture surface location, fracture configuration, angulation and callus formation were adapted from Baur et al. [22]. The keel bone was divided into thirds (cranial, middle, and caudal), and keel bone fractures were localized to one of these anatomical regions (Fig 1). Additionally, the surfaces of the bone involved by the fracture (cranial, caudal, dorsal, ventral) were recorded and the fractures characterized as described below (see also Fig 2, as well as S1 Fig). A keel bone was classified as fractured if a step formation was seen at the bone surface or if a fracture gap was present. Alternatively, cortical new bone formation associated with a radiopaque line running towards or all the way through to the opposite cortical surface were classified as chronic fissures and fractures, respectively. When a step formation was present, the fracture was classified as displaced and the direction of displacement was noted, while a fracture was classified as non-displaced if no step formation was present.

The degree of displacement was subjectively recorded as mild, moderate, or marked (S2 Fig). If the fracture resulted in an angular deviation of the long axis of the keel bone, the direction of the angulation was recorded. Fractures were further classified as complete or incomplete. For a complete fracture, the bone was broken through completely, separating the bone into two or more pieces—i.e., there was a disruption of two opposing cortices of the bone. For an incomplete fracture, the bone was not broken through completely and thus only a single cortex was disrupted, while the opposing one was intact. The complete fractures were subdivided into transverse, oblique, butterfly, and comminuted depending on the fracture configuration. Transverse fractures occurred at an angle perpendicular to the long axis of the keel bone, while fractures were classified as oblique when the angle of the fracture was greater than 10 degrees to the long axis of the keel bone. A fracture was classified as a butterfly fracture when two oblique fracture lines met to create a triangular fragment between the proximal and distal fracture fragments, while other fractures with two or more fracture fragments were classified as comminuted fractures. A lesion was classified as a folding fracture when there was a deformity of the bone, with the appearance of the bone folding in on itself, while a fracture was classified as a chip fracture when a small bone fragment was separated from the keel bone. For each radiograph, the degree of keel bone ossification was classified as either incomplete or complete. Keel bone ossification was classified as incomplete when the caudal margins of the keel bone were irregular, and the tip of the keel bone could either not be visualized or was seen as a soft tissue opaque structure. The keel bone was classified as completely ossified when the entire bone could be visualized as a smoothly marginated, mineral opaque structure. The binary classification of ossification (complete/incomplete) was considered more suitable than measuring the proportion of ossified keel, due to motion unsharpness and/or superimposition hampering accurate measures of the non-ossified part of the keel. The degree of callus formation associated with the fractures, as well as soft tissue swelling around the fractures was noted subjectively as mild, moderate, or marked (S3 and S4 Figs).

Fig 2 shows a flow chart of radiological characterization of fractures and the criteria for further dividing into incomplete or complete fractures, and their configuration. For further fracture characteristics we refer to S1 Fig. Strictly speaking, incomplete fractures can also have a transverse or oblique configuration, however only the complete fractures were given a configuration in the current study.

### Descriptive statistics

Data on flock laying percentage, flock egg weight, flock feed and water intake and flock mortality were retrieved from the farmer. Data on mean hen weight on flock level was retrieved from the farmer by repeated weighing on digital hanging scales placed in the barn. A summary of flock characteristics as well as descriptive statistics of the study sample are shown in Table 1. Data was recorded in Excel spread sheet [37] and later transferred to STATA [38] for descriptive statistics. Relative frequency of KBF, the different fracture morphologies, fractures in each anatomical location, and fully ossified keel bones, were calculated for each visit (i.e. each age group). Confidence intervals, with a probability limit of 95%, were calculated for KBF prevalence in each age group. The degree of ossification for different age groups was explored visually in several plots.

## Results

For descriptive statistics at hen level, we will refer to the study unit as “a hen”, although strictly speaking it is the combination of hen and time point. As all hens were released back in the flock at the end of each visit, the same hen might have been sampled more than once although not at the same sampling occasion (except for euthanized hens for the histological examination). No hens had to be excluded due to stress, nor immediately euthanized due to lethargy or trauma. The total study sample across all visits consisted of 330 radiographed hens. Of these, 190 hens had one or more KBF, with the majority (67%) detected in the last five visits (41–57 WOA). The number of KBF per keel bone varied from 1–5 fractures, where 105/190 hens had two or more KBF, resulting in altogether 344 KBF across all visits. For descriptive statistics of fracture characteristics we refer to the study unit as “a fracture”.

### Fracture characteristics

Of the 344 KBF, 246/344 (71.5%) were complete and 98/344 (28.5%) were incomplete fractures (Table 2). Looking at the complete fractures’ configuration, 204/246 (82.9%) were transverse, 39/246 (15.9%) were oblique, two were a butterfly fracture and one comminuted fracture. Of the 39 oblique fractures, 29/39 (74.4%) had a craniodorsal-caudoventral direction and 10/39 (25.6%) had cranioventral-caudodorsal direction. One chip fracture was recorded. There were 64/344 (18.6%) fractures recorded as folding fractures. Of the folding fractures 61/64 (95.3%) were transverse, 1/64 (1.6%) were oblique and 2/64 (3.1%) were incomplete.

**Table 2:** Overview of fracture completeness (n = 344 fractures), the location on the keel and frequency in each age group.

		Incomplete	Complete
Fracture completeness, n (%)		98 (28%)	246 (72%)
Number of complete and incomplete fractures in each keel location			
Cranial keel		24	3
Mid keel		3	0
Caudal keel		71	243
Number of complete and incomplete fractures in each age group			
	Weeks of age		
	17	0	0
	20	1	0
	25	3	6
	28	1	10
	32	8	18
	36	6	28
	41	17	17
	45	11	33
	49	15	36
	53	22	40
	57	14	58

**Fig 3:**
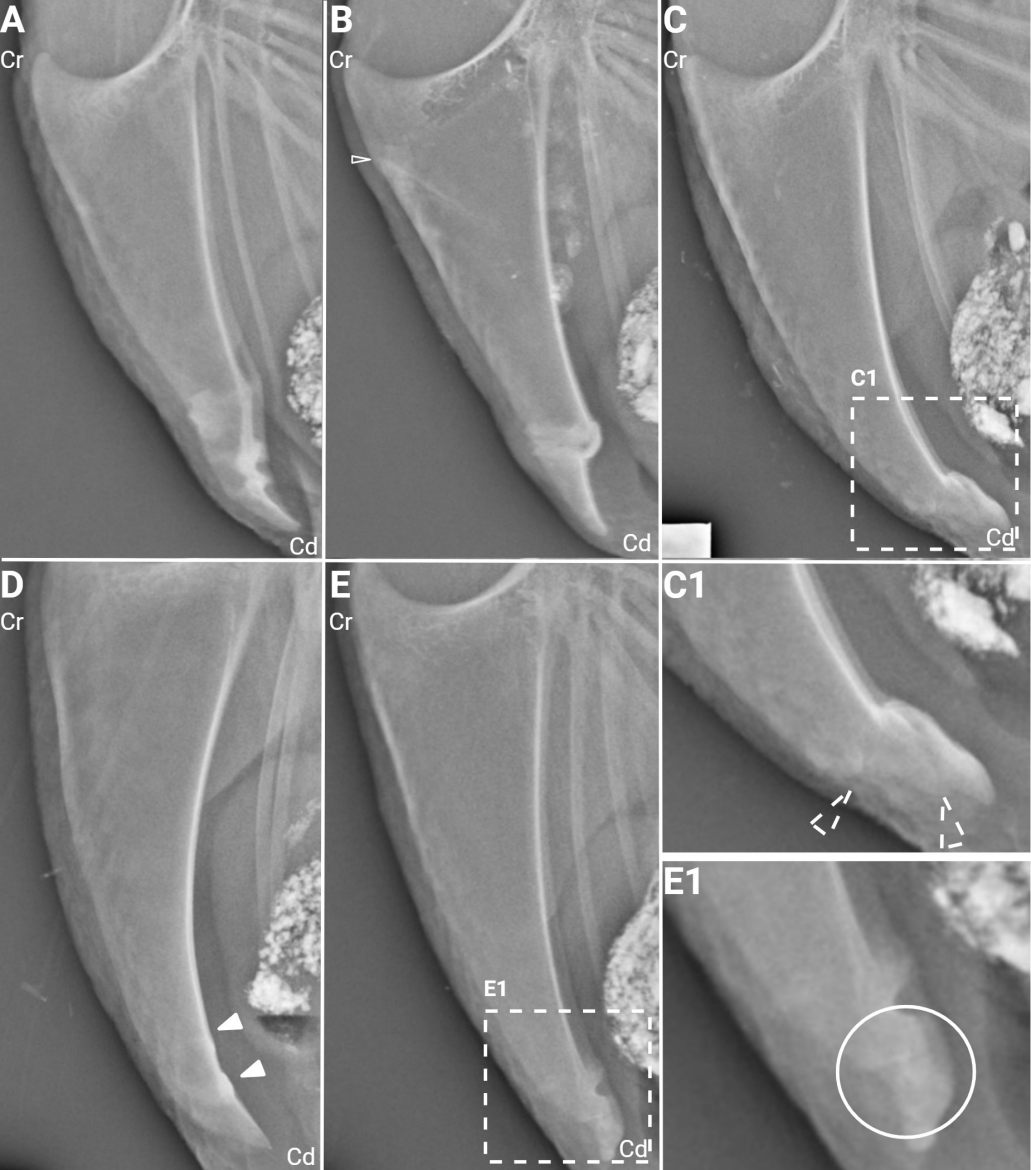
Fracture types. Figure showing different types of fractures. Cr = cranial, Cd = caudal (A) Three complete fractures in the caudal third of the keel, all with transverse configuration. (B) A complete fracture in the caudal third of the keel with oblique configuration. An incomplete fracture can be seen in the cranial third of the keel (hollow arrowhead). (C) A butterfly fracture in the caudal third of the keel. (C1) The butterfly fracture in C, enlarged. Two oblique fractures (dotted arrowheads) form a triangular fragment; the butterfly fracture. (D) Two incomplete fractures in the caudal third of the keel, appearing on the dorsal side of the keel (filled arrowheads). (E) Two complete fractures in the caudal third of the keel, with transverse configuration. The more caudal fracture is an example of a folding fracture. (E1) The folding fracture in E, enlarged and embedded in a circle. Created with BioRender.com.

An example of each fracture type is shown in Fig 3.

The majority of the fractures 314/344 (91.3%) appeared in the caudal third of the keel bone, followed by the cranial third 27/344 (7.8%) and mid keel 3/344 (0.9%). The majority of the fractures in the caudal third were complete fractures (243/314, 77.4%), whilst the majority of the fractures in the cranial third were incomplete fractures (24/27, 88.9%) (Table 2).

The fracture configuration of the complete fractures in the caudal third consisted mainly of transverse fractures (203/243, 83.5%) followed by oblique fractures (37/243, 15.2%). The two butterfly fractures and the one comminuted fracture appeared on the caudal third.

**Fig 4:**
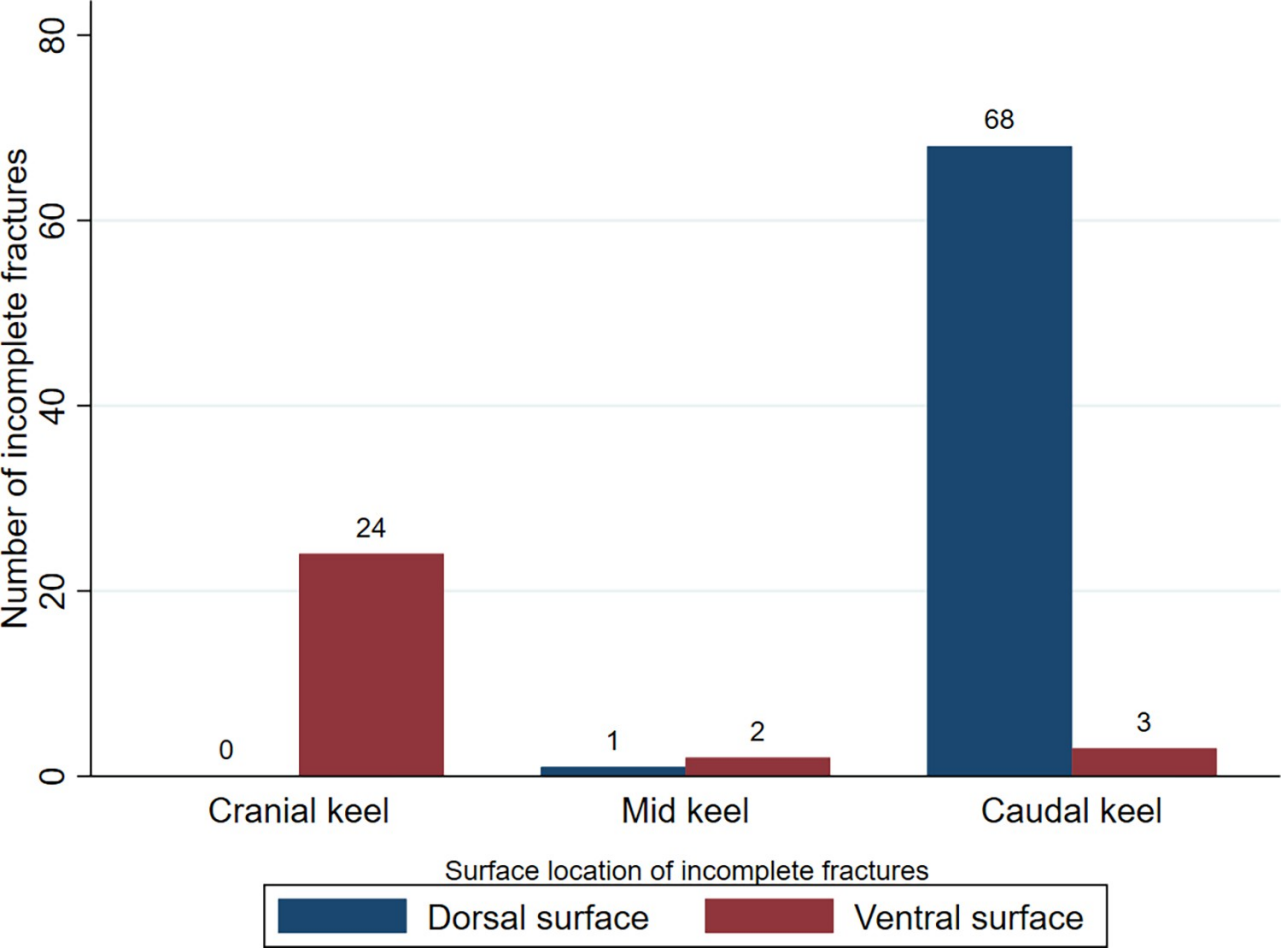
Surface location of incomplete fractures. Figure showing the number of incomplete fractures (n = 98) in each surface location (dorsal or ventral) and in each anatomical location (cranial-, mid- or caudal keel).

All the incomplete fractures on the cranial third appeared on the ventral side of the keel, while the majority of the incomplete fractures on the caudal third appeared on the dorsal side of the keel (Fig 4). The chip fracture, which is an incomplete fracture, appeared on the dorsal side on the caudal third of the keel. Fracture angulation was almost exclusively seen on the caudal third of the keel, with nearly even distribution between dorsal and ventral angulation (35 and 33%, respectively).

Most fractures had no soft tissue swelling (263/344, 76.5%), followed by mild soft tissue swelling (75/344, 21.8%) and moderate soft tissue swelling (6/344, 1.7%). The pattern observed for soft tissue swelling was the same within complete and incomplete fractures, as well as transverse and oblique fractures. Marked soft tissue swelling was not recorded for any fractures.

Callus was seen in 224/344 (65.1%) of the fractures, in which the majority was categorized as mild (152/344, 44,2%). Marked callus was exclusively seen in the caudal third of the keel bone and in three fracture types: four transverse fractures, four oblique fractures and the one butterfly fracture.

The majority of the fractures had no displacement (249/344, 72.4%), followed by mild (78/344, 22.7%), moderate (10/344, 2.9%) and marked (7/344, 2.0%). The most common direction of displacement was dorsal (60/344, 17.4%). The marked displacements appeared for the first time at 45 WOA.

### KBF and age

**Fig 5:**
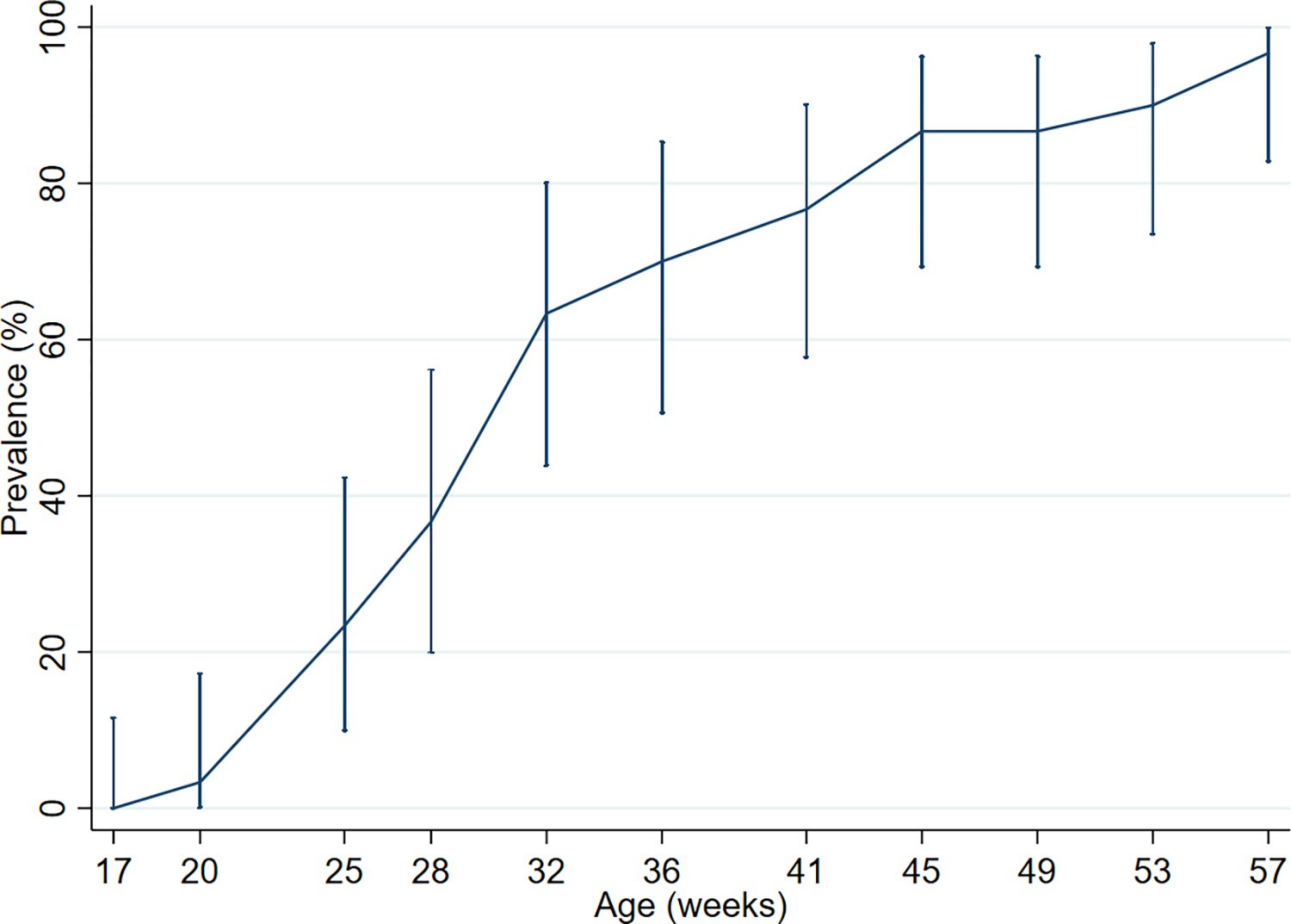
Keel bone fractures in each age group. Figure showing the prevalence of keel bone fractures (KBF), with 95% CI, in each age group. Confidence interval for 17 weeks of age is one sided 97.5% confidence interval.

The prevalence of KBF increased with age, except between 45 and 49 WOA (Fig 5). The highest increase was between 28 and 32 WOA (Fig 5). Table 3 shows the frequency of KBF in cranial-, mid-, and caudal third of the keel, in each age group.

**Table 3:** Number of fractures (n = 344) on cranial-, mid- and caudal keel, in each age group.

Week of age	Cranial keel	Mid keel	Caudal keel
17	0	0	0
20	0	0	1
25	1	0	8
28	0	0	11
32	2	0	24
36	2	0	32
41	2	0	32
45	2	1	41
49	5	0	46
53	5	1	56
57	8	1	63

**Fig 6:**
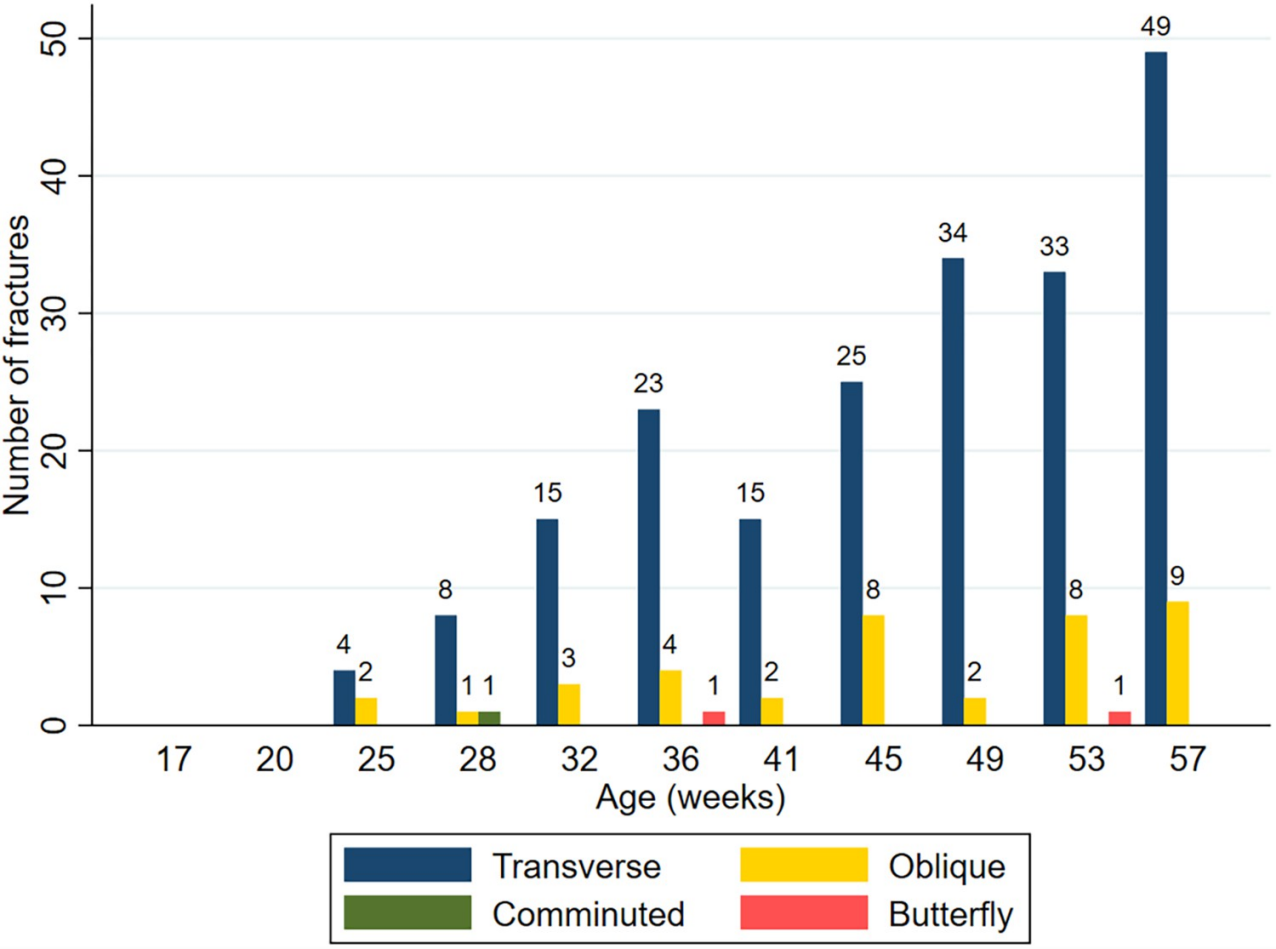
Fracture configuration. Figure showing configuration of complete fractures (n = 247) in each age group.

Complete fractures dominate in each age group, except at 41 WOA where fractures were evenly distributed between complete and incomplete fractures (Table 2). Fracture configuration in each age group is shown in Fig 6. Transverse fractures were observed as the most common configuration across all age groups.

The folding fractures were observed in alle age groups from 28 WOA. The number of folding fractures increased with age, however the relative frequency of folding fractures within each age group remained relatively constant, ranging from 17–20% of all fractures in each age group from first occurrence at 28 WOA.

**Fig 7:**
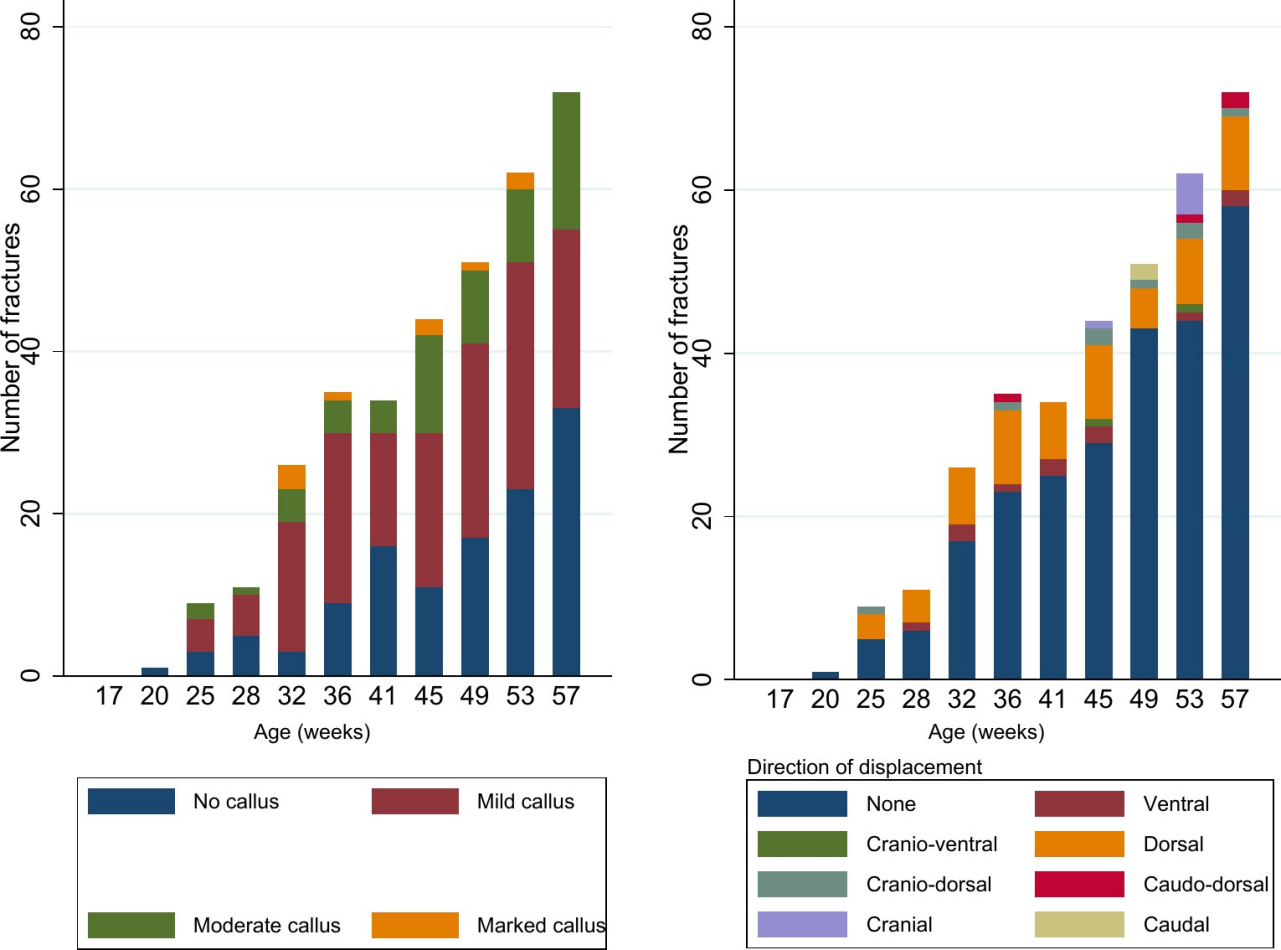
Callus grading and direction of displacement. Figure showing number of fractures’ callus grading in each age group (left panel) and number of fractures’ displacement direction in each age group (right panel).

The relative frequency of the different callus grading in each age group is shown in Fig 7. No callus and mild callus dominate in each age group. The earliest appearance of marked callus was at 32 WOA (Fig 7). When looking at the fractures with displacement, the relative frequency of the different displacement directions gets more complex later in the production cycle, as seen in Fig 7.

### Ossification

**Fig 8:**
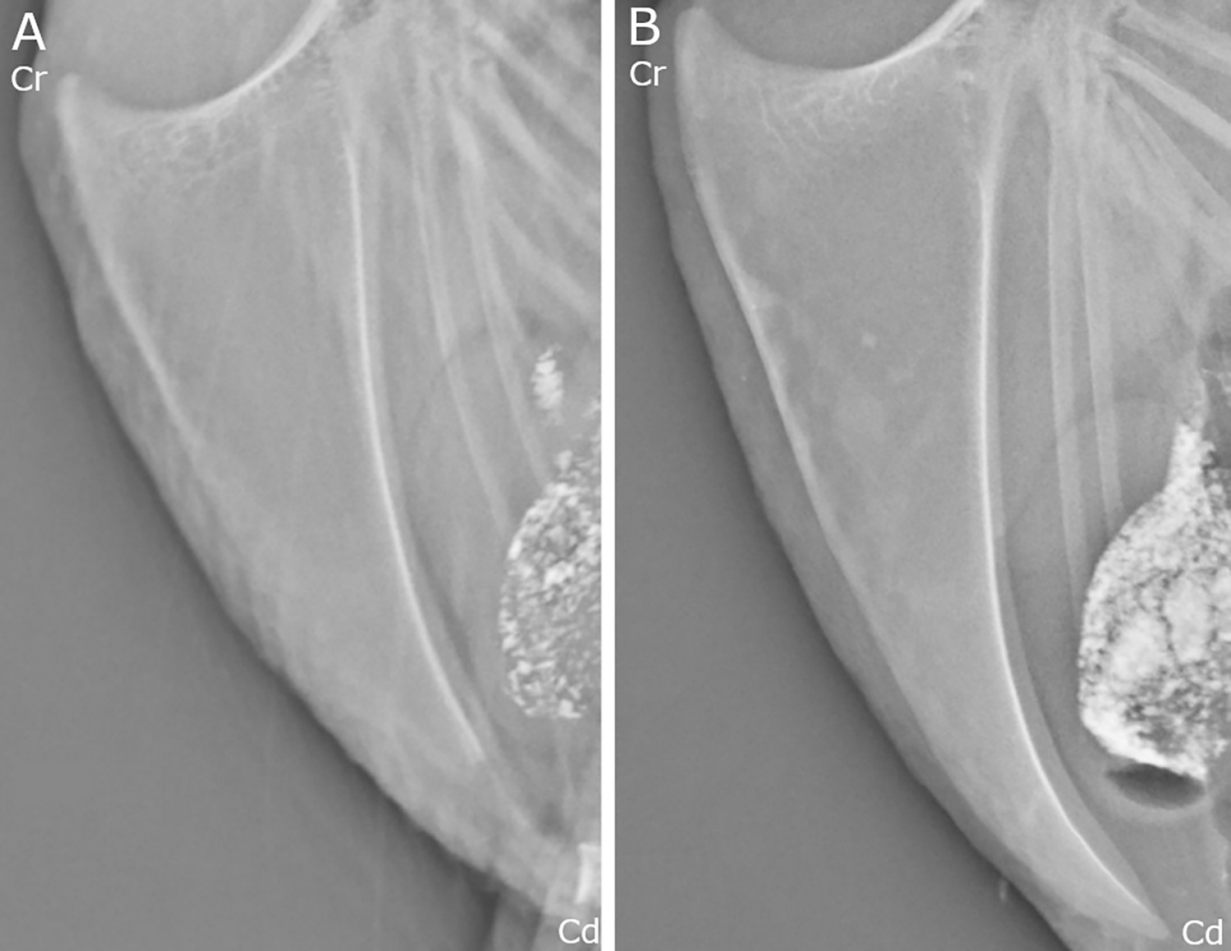
Ossification. A Figure showing an example of a non-ossified keel and fully ossified keel. Cr = cranial, Cd = caudal. (A) A keel from a 17 weeks of age (WOA) hen, with indistinct and mildly irregular margins in the caudal third of the keel, indicating that the keel is not fully ossified. (B) a keel from a 36 WOA hen with smooth and sharply defined margins along the whole keel, indicating a fully ossified keel.

Based on the radiographic evaluation, the keel bone was fully ossified for all hens from 32 WOA and older. At 17 and 20 WOA, no hens had fully ossified keel bones. However, there were two 20 WOA hens in which the ossification could not be evaluated due to poor image quality. An example of a non-ossified keel and a fully ossified keel is shown in Fig 8.

Keel bone fractures appeared in non-ossified keels. At 20 WOA the one chip fracture appeared in the caudal third in a non-ossified keel bone, although the fracture was within the ossified region of the bone. At 25 WOA, there were six hens with KBF in the caudal third, of which four did not have a fully ossified keel bone. At 28 WOA, 11 hens had KBF in the caudal third where two of them did not have a fully ossified keel bone.

## Discussion

In this study we aimed to provide a detailed description of fracture morphology, fracture occurrence, and keel bone ossification for different age groups, using radiography. There was a high frequency of simple fractures without displacement and soft tissue swelling. Additionally, fracture characteristics differed between the cranial and caudal third of the keel.

### Fracture types

Fracture morphology is affected by the force of impact, additionally the physiological qualities of the bone and anatomical features will affect the bone’s ability to resist forces [17, 18, 39].

The majority of fractures were complete. This is in line with Baur et al. [22], who used radiography to describe KBF characteristics in a longitudinal experimental study. Thøfner et al. [15] also reported the majority of fractures as simple complete fractures in a study on KBF histology. The most common configuration of the complete fractures in the current study was transverse fractures, also reported by Baur et al. [22]. In human forensic medicine, transverse fractures are described to occur when a bone bends, experiencing both compressive and tensile stress due to direct or indirect force [18, 40]. A transverse fracture from a high impact force will commonly result in a displacement of the fracture and, in the acute phase, swelling of the surrounding tissue [40]. A majority of the transverse fractures in the current study had no displacement and little to no soft tissue swelling, which can imply that they did not result from a high impact force. However, the lack of soft tissue swelling can also be explained by the fact that most of the fractures were chronic fractures with signs of remodeling, which can be associated with mild to no soft tissue swelling on radiography. Forces applied to the bone resulting in a fracture might be external or internal. In a laying hen, the pull from muscles on a weakened bone or repeated pressure from inside the hen could be possible internal forces like repeated pressure from egg laying or pectoral muscles pulling the keel bone during muscle contraction, as also discussed by Thøfner et al. [2, 15, 41]. We recorded one chip fracture in the current study. This fracture was observed in a 20 WOA hen in the caudal third of the keel. A chip fracture occurs when a ligament or a tendon pulls off a small fragment of the bone. This commonly occurs when the bone structure is weaker than the ligament or tendon. This supports the possibility that some KBF occur due to pectoral muscles pulling on a weakened keel. Oblique fractures were the second most common fracture configuration in the current study. In long bones in humans, an oblique fracture usually results from a combination of compression or bending, together with rotation [18]. In the study by Baur et al. [22], oblique fractures were also the second most common fracture configuration (43% of all fractures). The origin of force causing the oblique fractures in the current study is uncertain.

There was one comminuted fracture and two butterfly fractures detected in the current study. These fracture types tend to be a result of a high force to the bone [17, 18, 40]. Thus, with an aviary housing system, they could arise from high impact collisions into the housing system, e.g. into the tiers and/or perches as suggested by other studies [10, 28, 42]. The low frequency in the current study is in agreement with both Baur et al. [22] and Thøfner et al. [15], indicating that high impact collisions are unlikely to be the primary cause of most KBF.

Incomplete fractures were more common in the cranial third compared to the other parts of the keel in the current study. Baur et al. [22] did not report on incomplete fractures specifically, however they reported non-fracture lesions. One of those lesions were described as “new bone formations without a fracture gap” (15%), which was recorded as a chronic fissure (i.e. incomplete fracture) in the current study. Fissures can later develop into complete fractures [39]. In the study by Baur et al. [22], 55% of the non-fracture lesions, including new bone formation, developed into fractures at a later stage. This was not possible to examine in the current study as we sampled new birds at each timepoint. Interestingly, on the keel’s cranial third, all the incomplete fractures appeared on the ventral surface whilst most of the incomplete fractures on the caudal third appeared on the dorsal surface. This finding might indicate a different etiology for the incomplete fractures in the cranial versus the caudal third of the keel; the ventral appearance suggests an external impact while internal forces are likely involved for the dorsal appearance on the keel. Further longitudinal studies with a larger sample size are warranted to confirm this.

In the current study, 64 fractures had an appearance that resembled folding fractures. This was observed both in the cranial and caudal third of the keel. Folding fractures were more frequent in the older hens from 49–57 WOA, though the proportion of folding fractures, relative to all fractures, remained the same in each age group from first appearance. Folding fractures are seen e.g. in cats and dogs with disorders of calcium metabolism, suggesting that bone pathology such as calcium depletion may also play a role in KBF pathogenesis. Studies have reported associations between bone mineral density, bone strength, KBF and keel bone deviations, i.e. increased bone strength and bone mineral density decreases the risk of overall damage to the keel [3, 11, 43–46]. The egg laying related changes in bone metabolism may alter the maturation of the keel, which is not fully ossified at sexual maturity, and early onset of lay has been suggested as a risk factor for KBF [47, 48]. Dunn et al. [49] also discusses the importance of ensuring adequate bone development before hens enter lay. More knowledge on hen level risk factors are still in demand and should be pursued in longitudinal studies of individual hens.

### Fractures and age

In the current study we reported on KBF prevalence (i.e. birds with at least one fracture) in each age group instead of KBF incidence, as we did not follow individual birds. There is a chance that the same hen might have been sampled again at a later visit, nonetheless the likelihood of choosing the same hen among 7500 hens is low. The youngest hen with a KBF was 20 WOA. Previous studies have shown that KBF starts appearing around 20 to 25 WOA, and some as early as 16 WOA in both cage and non-cage systems [13, 14, 22]. The prevalence of KBF increased with each age group, not surprisingly as KBF accumulates over time. Increase in KBF prevalence with age has also been reported by Eusemann et al. [21] in their longitudinal study radiographing laying hens at 35, 51 and 72 WOA. In the current study, the greatest increase in KBF prevalence was observed between 28 and 32 WOA. This is in line with Baur et al. [22] who reported that most new fractures occurred between 28 and 37 WOA. Other studies have reported an increasing incidence of KBF from 20 WOA and later decreasing after 50 WOA [13, 14, 28]. The reason for this pattern in KBF incidence is still uncertain and warrants further investigation.

### Callus formation and soft tissue swelling

In the current study 65% of all fractures had callus formation, however the majority had mild callus formation (44.2%) and 35% had no callus formation at all. Baur et al. [22] reported callus formation in 76% of all fractures. In a study on histological characteristics of KBF in commercial layers, the proportion of hens with no callus was higher in 35 WOA hens compared to hens older than 75 WOA, and of the hens with callus formation the majority had minimal callus [15]. Callus formation has also been reported higher in laying hens from non-cage system compared to caged systems [2, 15]. A common diagnostic method of KBF is palpation of the keel bone. Using palpation, a callus is essential for detecting old fractures. Palpation is a diagnostic method with low accuracy, especially for detecting fractures with minimal callus, incomplete fractures and/or fractures on the dorsal surface of the keel bone [2, 23, 30, 50, 51]. The proportion of fractures without callus formation found in the present study supports the possibility of underestimation of fracture prevalence recorded through palpation alone.

Most of the fractures (76.5%) had no soft tissue swelling. This could be due to the age of the fractures, as soft tissue swelling diminishes as the fracture gets older. Since we did not follow the same hen at each visit, we could not separate between recent and older fractures. Another explanation could be the challenges in evaluating soft tissue swelling around the keel bone in a latero-lateral view. Soft tissue swelling around the dorsal surface of the keel bone is challenging due to superimposition of internal organs. However, the lack of soft tissue swelling has been reported previously [15, 22, 23]. Histological samples of KBF revealed little to no inflammation and hemorrhage in the fracture gap and surrounding soft tissue in a study by Thøfner et al. [15]. The lack of soft tissue swelling and inflammation in and around the fractures could indicate stress fractures, i.e. fractures caused by continuous stress on the bone.

### The caudal third of the keel and its ossification

The caudal third of the keel bone was the most common site for fractures (91.3%). This is in line with several other studies [2, 22, 29, 30, 52]. The caudal third is prominent with less soft tissue than the mid and cranial third, where larger muscle area might absorb the impact more effectively. It has also been reported that the caudal third of the keel bone has less bone mineral density than the cranial third [43]. This could also explain why most of the fractures on the cranial third and mid third of the keel were incomplete fractures, whereas complete fractures dominated in the caudal third of the keel. The comminuted and the butterfly fractures occurred on the caudal third as well. However, the caudal third is also the last part of the keel bone to ossify and existing literature suggests that ossification in the keel is not complete until at 40 WOA [27]. In the current study we reported the ossification to be complete somewhat earlier than this, at 32 WOA. However, superimposition of surrounding tissue and occasionally of the knees limits the possibility to fully measure the ossification. It is therefore expected that a more accurate tool like histology can identify ossification at a later stage, than what is possible with radiography. It is known that KBF start appearing before 40 WOA, also shown in the current study, and that the hypertrophic zone in endochondral ossification is a weak spot and often involved in fractures in growing individuals [31, 32]. Fractures in the caudal third might be a result of continuous stress on that area originating from inside the hen or from muscle contraction from wing flapping, as suggested by Thøfner et al. [2, 15, 41]. In the current study we report that in some hens KBF appeared before the keel was fully ossified, however not in the hypertrophic zone of the ossification. As we do not know the exact time of fracture, we do not know if the KBF in the fully ossified keels occurred before the keel was ossified. Further research is needed to explore the relationship between ossification and risk of KBF.

### Study limitations

Radiographs were taken in a single latero-lateral view. Preferably, a minimum of two radiographs at 90-degree angles should be taken to evaluate a fracture. A single view can lead to misinterpretation and underestimation of fractures. However, the dorsoventral view of the keel bone will give limited information due to superimposition of the keel bone with the spine and internal organs. This shortcoming has also been discussed in previous studies on radiography of the keel bone [21, 22]. A previous study compared the diagnostic accuracy of portable radiography relative to dissection and found that both the sensitivity and specificity was high [24]. Alternatively, using a CT scan will give a complete and detailed overview of fracture characteristics, as well as information on ossification. This is however challenging to conduct in a commercial setting. The intra-observer repeatability was not systematically evaluated in the current study. A high intraclass correlation coefficient was found in a previous study using a radiographic protocol for assessment of KBD [19].

It is important to emphasize that the study sample is small and solely from a single flock. This implies a cautious approach to generalization of the reported prevalence and fracture characteristics in each age group.

Nevertheless, this study sheds light on KBF characteristics in aviary commercial settings. We report a frequent occurrence of simple complete fractures without displacement and soft tissue swelling, as well as incomplete fractures instead of complex fractures as would be expected by high impact forces [53]. Additionally, we pose a hypothesis that the etiology behind fractures occurring in the cranial third might differ from fractures in the caudal third of the keel bone. We suggest that external forces might be a more important cause in the cranial third, whilst internal forces might be more important for fractures on the caudal third.

In order to fully understand the etiology of KBF, further longitudinal studies are needed with frequent records in individual hens. While ossification of keel bone can be measured in radiographic images, a more accurate tool like histology is preferable in order to describe the ossification and bone maturation in detail.

## Conclusion

Most of the fractures in the present study were complete (71.5%) while 28.5% were incomplete. The most common configuration was transverse fractures (82.9%) followed by oblique fractures (15.9%). A clear difference in incomplete fracture appearance was seen in the cranial and caudal third of the keel bone. Based on this, we suggest that external impact might be a contributing factor for the cause of fractures in the cranial third, while internal forces might be a more important part of the etiology behind fractures in the caudal third of the keel. Typical high-force impact fractures were infrequent. Fractures resembling folding fractures were seen, indicating a possible preexisting bone pathology or the dilemma of considerable calcium demand in both eggshell formation as well as in ossification and maturation of the skeleton, including the keel bone. Radiographic evaluation showed that the keel bone was fully ossified in all studied hens at 32 WOA and older in a commercial layer flock.

## Supporting information

S1 Fig Radiological fracture characterization. A flow chart showing further characterization of fractures, in addition to their completeness and configuration as seen in Fig 2. Created with BioRender.com.
(TIFF)

S2 Fig Fracture displacement. Different degrees of displacemet. Cr = cranial. Cd = caudal. (A) No displacement in two incomplete fractures. (B) Mild displacement in a butterfly fracture. (C) Moderate displacement in an oblique fracture. (D) Marked displacement in a transverse fracture in the caudal third. Additionally, a mild displacement can be seen in the incomplete fracture in the cranial third. The categorization was made subjectively, based on experience. An objective classification based on measurements in the images was not attempted, as measurements in a projection imaging modality such as radiography is dependent on exact, standardized projections which are challenging to obtain in awake animals in a barn setting.
(TIF)

S3 Fig Callus formation. Different degrees of callus formation. Cr = cranial. Cd = caudal. (A) No callus formation in a transverse fracture. (B) mild callus formation in an incomplete fracture on the ventral surface of the keel. (C) moderate callus formation in three transverse fractures. (D) marked callus formation in an oblique fracture. The categorization was made subjectively, based on experience. An objective classification based on measurements in the images was not attempted, as measurements in a projection imaging modality such as radiography is dependent on exact, standardized projections which are challenging to obtain in awake animals in a barn setting.
(TIF)

S4 Fig Soft tissue swelling. Different degrees of soft tissue swelling. Cr = cranial. Cd = caudal. (A) No soft tissue swelling around two transverse fractures. (B) mild soft tissue swelling around a transverse fracture. (C) moderate soft tissue swelling around a transverse fracture. No marked soft tissue swelling was recorded in the study. The categorization was made subjectively, based on experience. An objective classification based on measurements in the images was not attempted, as measurements in a projection imaging modality such as radiography is dependent on exact, standardized projections which are challenging to obtain in awake animals in a barn setting. For an acute traumatic fracture, there will be no remodeling of the fracture ends, i.e. the fracture margins will be sharp and well defined, and there will be no callus formation. If such a fracture results from an external force, concurrent trauma to the overlying soft tissues, and thus a soft tissue swelling would be expected. Hemorrhage from the fracture ends will often also contribute to the soft tissue swelling. Fractures with radiographic evidence of remodeling of the fracture ends will either be chronic traumatic fractures or acute fractures where pre-existing bone pathology has been present. Some soft tissue swelling may still be present in chronic fractures, particularly if there is continued motion at the fracture site. However this swelling will typically be milder than in the acute phase.
(TIF)

S1 Data Data from radiological evaluation. (XLSX)
