# Supplementary figures and images for: Fracture morphology and ossification process of the keel bone in modern laying hens based on radiographic imaging
Source: PLoS One. 2024 Oct 29;19(10):e0312878. doi: 10.1371/journal.pone.0312878 (PMC11521247; doi:10.1371/journal.pone.0312878)

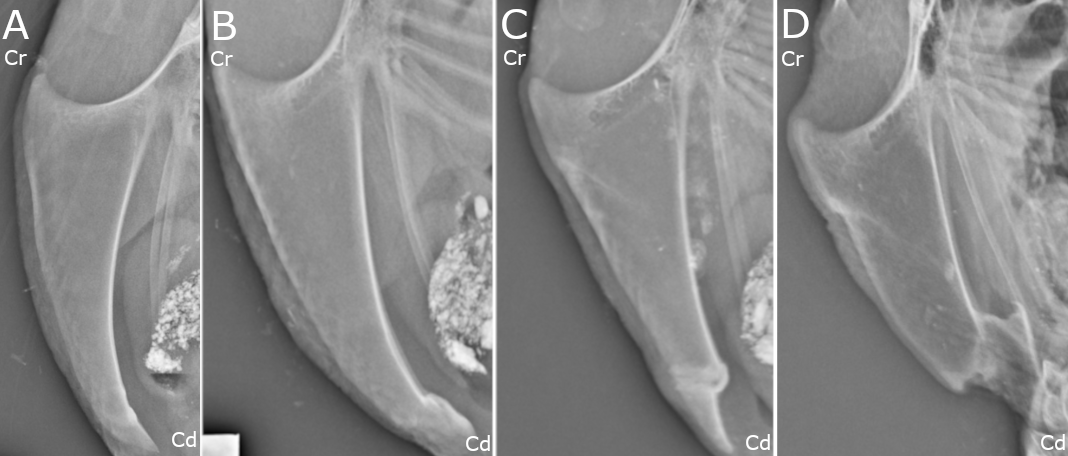

Supplement: S2 Fig — Different degrees of displacemet. Cr = cranial. Cd = caudal. (A) No displacement in two incomplete fractures. (B) Mild displacement in a butterfly fracture. (C) Moderate displacement in an oblique fracture. (D) Marked displacement in a transverse fracture in the caudal third. Additionally, a mild displacement can be seen in the incomplete fracture in the cranial third. The categorization was made subjectively, based on experience. An objective classification based on measurements in the images was not attempted, as measurements in a projection imaging modality such as radiography is dependent on exact, standardized projections which are challenging to obtain in awake animals in a barn setting. (TIF) [file pone.0312878.s002.tif]

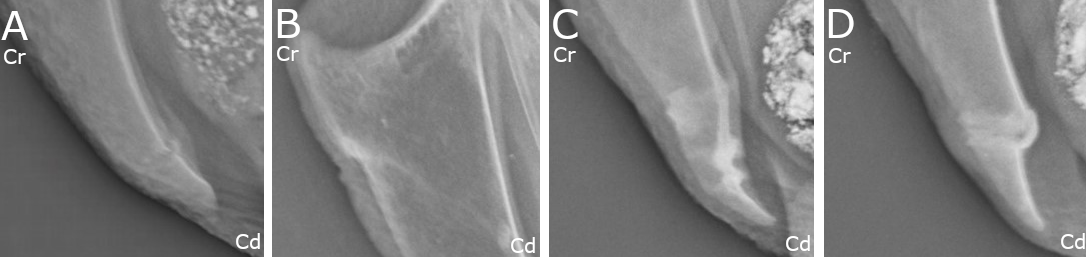

Supplement: S3 Fig — Different degrees of callus formation. Cr = cranial. Cd = caudal. (A) No callus formation in a transverse fracture. (B) mild callus formation in an incomplete fracture on the ventral surface of the keel. (C) moderate callus formation in three transverse fractures. (D) marked callus formation in an oblique fracture. The categorization was made subjectively, based on experience. An objective classification based on measurements in the images was not attempted, as measurements in a projection imaging modality such as radiography is dependent on exact, standardized projections which are challenging to obtain in awake animals in a barn setting. (TIF) [file pone.0312878.s003.tif]

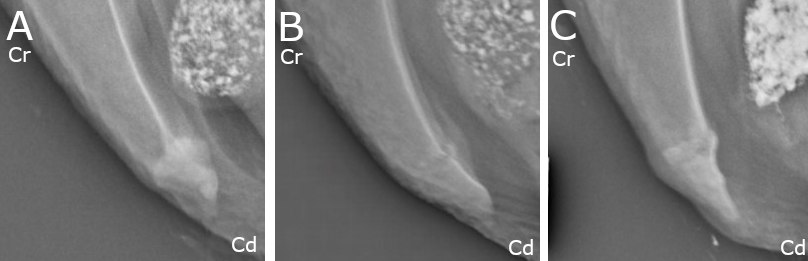

Supplement: S4 Fig — Different degrees of soft tissue swelling. Cr = cranial. Cd = caudal. (A) No soft tissue swelling around two transverse fractures. (B) mild soft tissue swelling around a transverse fracture. (C) moderate soft tissue swelling around a transverse fracture. No marked soft tissue swelling was recorded in the study. The categorization was made subjectively, based on experience. An objective classification based on measurements in the images was not attempted, as measurements in a projection imaging modality such as radiography is dependent on exact, standardized projections which are challenging to obtain in awake animals in a barn setting. For an acute traumatic fracture, there will be no remodeling of the fracture ends, i.e. the fracture margins will be sharp and well defined, and there will be no callus formation. If such a fracture results from an external force, concurrent trauma to the overlying soft tissues, and thus a soft tissue swelling would be expected. Hemorrhage from the fracture ends will often also contribute to the soft tissue swelling. Fractures with radiographic evidence of remodeling of the fracture ends will either be chronic traumatic fractures or acute fractures where pre-existing bone pathology has been present. Some soft tissue swelling may still be present in chronic fractures, particularly if there is continued motion at the fracture site. However this swelling will typically be milder than in the acute phase. (TIF) [file pone.0312878.s004.tif]
